# Chitosan Composites with Bacterial Cellulose Nanofibers Doped with Nanosized Cerium Oxide: Characterization and Cytocompatibility Evaluation

**DOI:** 10.3390/ijms24065415

**Published:** 2023-03-12

**Authors:** Valentina A. Petrova, Iosif V. Gofman, Natallia V. Dubashynskaya, Alexey S. Golovkin, Alexander I. Mishanin, Elena M. Ivan’kova, Dmitry P. Romanov, Albert K. Khripunov, Elena N. Vlasova, Alexandra V. Migunova, Alexander E. Baranchikov, Vladimir K. Ivanov, Alexander V. Yakimansky, Yury A. Skorik

**Affiliations:** 1 Institute of Macromolecular Compounds of the Russian Academy of Sciences, Bolshoi VO 31, St. Petersburg 199004, Russia; 2 Almazov National Medical Research Centre, Akkuratova 2, St. Petersburg 197341, Russia; 3 Institute of Silicate Chemistry of the Russian Academy of Sciences, Adm. Makarova emb. 2, St. Petersburg 199034, Russia; 4 Department of Microbiology, Faculty of Biology, St. Petersburg State University, Universitetskaya emb. 7-9, St. Petersburg 199034, Russia; 5 Kurnakov Institute of General and Inorganic Chemistry of the Russian Academy of Sciences, Leninskii 31, Moscow 119071, Russia

**Keywords:** nanocomposites, biopolymers, bacterial cellulose, chitosan, ceria nanoparticles, stem cells’ proliferation

## Abstract

In this work, new composite films were prepared by incorporating the disintegrated bacterial cellulose (BCd) nanofibers and cerium oxide nanoparticles into chitosan (CS) matrices. The influence of the amount of nanofillers on the structure and properties of the polymer composites and the specific features of the intermolecular interactions in the materials were determined. An increase in film stiffness was observed as a result of reinforcing the CS matrix with BCd nanofibers: the Young’s modulus increased from 4.55 to 6.3 GPa with the introduction of 5% BCd. A further increase in Young’s modulus of 6.7 GPa and a significant increase in film strength (22% increase in yield stress compared to the CS film) were observed when the BCd concentration was increased to 20%. The amount of nanosized ceria affected the structure of the composite, followed by a change in the hydrophilic properties and texture of the composite films. Increasing the amount of nanoceria to 8% significantly improved the biocompatibility of the films and their adhesion to the culture of mesenchymal stem cells. The obtained nanocomposite films combine a number of favorable properties (good mechanical strength in dry and swollen states, improved biocompatibility in relation to the culture of mesenchymal stem cells), which allows us to recommend them for use as a matrix material for the culture of mesenchymal stem cells and wound dressings.

## 1. Introduction

Natural polysaccharides are promising materials for biomedical applications due to their biocompatibility, biodegradability, non-toxicity, low immunogenicity, and biological activity. The various polysaccharide-based polymer composites can be obtained in the form of films, sponges, electrospun mats, hydrogels, and nano- and microparticles. The design of polymer composites involves a combination of polymers with different chemical structures and physical forms, as well as a combination of polymers with nanoscale modifiers [1]. Polymer composites of chitosan (CS) and bacterial cellulose (BC) that combine the structure-forming ability, mechanical properties, and hydrophilicity of BC with the antimicrobial properties of CS are considered promising [2]. 

CS is a polyaminosaccharide derived from the deacetylation of chitin; CS is biodegraded in the human body under the action of lysozyme to N-acetylglucosamine and glucosamine, natural metabolic products [3]. CS has been widely used to design various tissue engineering structures [4,5]. A disadvantage of CS is the low strength of the formed films in the wet state. In order to produce materials with the enhanced complex of properties (the optimal combination of mechanical properties, surface charge, and biological activity), the strategy of introducing different nanofillers, including nanofibers of chitin or cellulose, into the CS matrix is widely used [6,7,8,9]. 

One of the most promising natural polymers for biomedical applications is BC, which is a linear, unbranched polysaccharide consisting of 1,4-glucopyranose units that is biosynthesized by microorganisms (e.g., *Komagataeibacter rhaeticus*) [10,11]. BC is characterized by a complex hierarchical structure and a high degree of crystallinity [12]. In BC, nanofibrils are highly oriented nanocrystals embedded in an amorphous matrix. These individual nanofibrils are further assembled to form highly oriented microfibrils or even fiber bundles, conferring exceptional mechanical properties to biological materials. Because of its structural similarity to the components of the extracellular matrix (e.g., collagen), BC is capable of interacting with biological tissues [13,14]. This complex of beneficial properties makes BC a promising biomaterial for the development of biocompatible matrices for tissue engineering [15,16,17,18,19,20]. However, the use of BC has a number of drawbacks, including the lack of antimicrobial properties, difficulty in regulating the pore size, and slow degradation [21]. 

The introduction of BC nanofibers into CS matrices can lead to the formation of composite materials with a number of valuable properties, such as improved mechanical properties, an optimized surface charge, and useful morphological features. Both the starting material and the methods of BC nanofiber isolation can influence the efficiency of nanofibrillation and the properties of the products, including morphology and crystallinity, which further affect the physical properties of the material. BC nanofibers obtained by the mechanical treatment of BC are typically characterized by both long length and high aspect ratio (length up to 500 µm, and thickness up to 50 nm). Since BC nanofibers have a large number of hydroxyl groups on their surface, and nanofibers isolated from BC have a high specific surface area, their surface can be easily functionalized and their interaction with the polymer matrix components can lead to a change in the polymer structure to form a nanocomposite with precisely tuned properties [9,12,22,23]. For example, Fernandes et al. [24] developed composites based on CS and BC nanofibers (5–40% of the CS weight) as a reinforcing agent. The surface morphology of the composite films showed the presence of a three-dimensional fibrillar network of BC. The observed crystallization of CS in the obtained nanocomposites is explained by the deposition of CS on the surface of the crystalline domains of BC nanofibers. Phisalaphong and Jatupaiboon [25] obtained composite films of BC with the addition of CS, which have excellent mechanical properties in wet and dry states, high water absorption and water retention capacity, and a high surface area. These materials have bacteriostatic and bactericidal activities.

Another important factor is the relationship between mechanical properties, geometry, and biological properties of scaffolds for tissue engineering. In this regard, the development of biocompatible composites containing nanoparticles is being considered [10,12,24,25,26]. Ceria nanoparticles are attractive inorganic fillers that strongly influence the physicochemical and biological properties of polymer systems. The introduction of ceria into the polymer matrix imparts specific biological activity to the composite and also changes its physical and mechanical properties; on the other hand, the polymer environment can regulate the properties of the nanoceria (e.g., reduce their toxicity). Thus, CeO2-containing polymeric nanocomposites combine the advantages and reduce the disadvantages of both polymer matrices and nanoparticles [27]. In addition, ceria has beneficial effects and provides cell and tissue protection through in vitro and in vivo inhibition of reactive oxygen species, suppression of cytokine levels, and reduction in inflammation [28,29,30,31].

In our previous studies, we obtained BC-based composites by impregnating the squeezed BC gel film with polysaccharide solutions containing citrate-stabilized ceria nanoparticles (CeONPs) [12] and CS-based composites containing CeONPs [32]. These studies have demonstrated the beneficial effects of CeONPs on the culture of human mesenchymal stem cells (MSCs), as evidenced by changes in stem cell behavior such as their migration, proliferation, and differentiation [12,32]. 

The next phase of our research was to develop a multi-component CS-BC-CeONP composite. This composite would combine the advantages of both previously developed scaffolds. Thus, the aim of this study was to obtain CS-based nanocomposites containing ensembles of nanoparticles with different architectures (disintegrated BC nanofibers and ceria nanoparticles), to study their interactions and the influence of the amount of nanoparticles present in the composite to modulate the physical and mechanical properties and network architecture of the nanocomposite, and to improve its biomedical potential for MSC culture.

## 2. Results and Discussion

### 2.1. Infrared Spectroscopy

**Figure 1:**
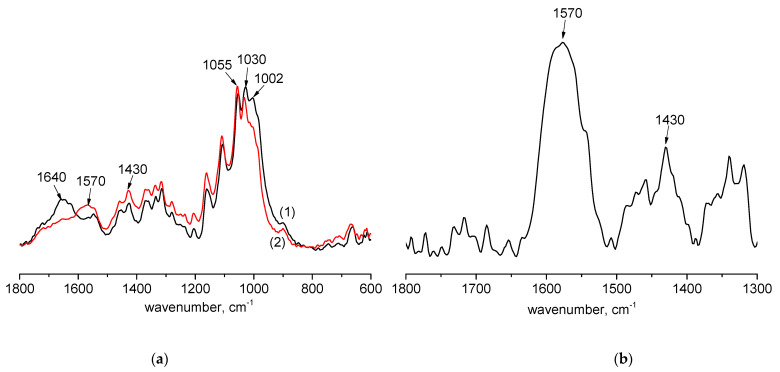
FTIR spectra: (**a**) the original bacterial cellulose (BC) (1) and disintegrated bacterial cellulose (BCd) (2); (**b**) difference spectrum of BCd and BC.

The comparative Fourier-transform infrared (FTIR) spectra of the original BC and disintegrated bacterial cellulose (BCd) (Figure 1a) show that in the spectrum of BCd, the 1570 cm^−1^ band appears and the 1430 cm^−1^ band changes (the band change is most obvious in the difference spectrum, Figure 1b). These bands correspond to the antisymmetric and symmetric vibrations of the –COO^−^ group. At the same time, a change in the shape and mutual intensities of the bands in the region of 1150–1000 cm^−1^ corresponding to the vibrations of the COC, C-C, C-OH, CH, and OH groups is observed. It can be assumed that during the preparation of disintegrated BCd, the BC molecules are degraded with the formation of terminal carboxylate groups.

**Figure 2:**
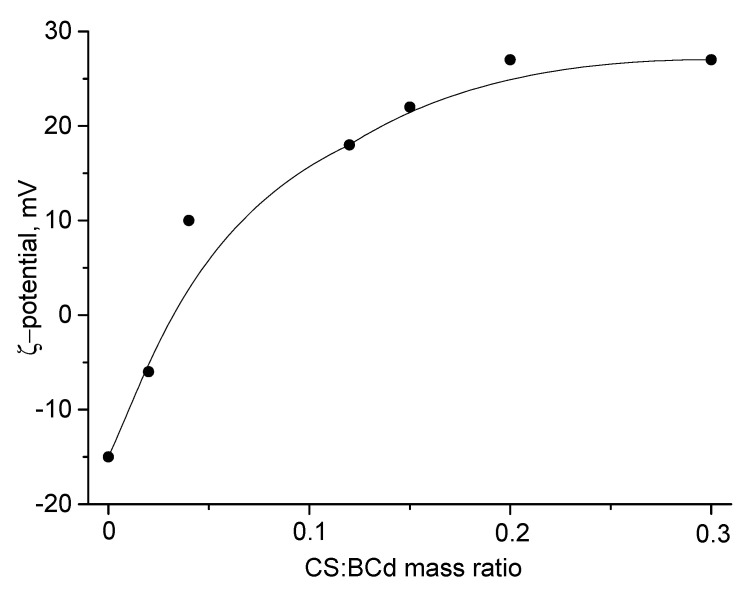
Changes in the ζ-potential of aqueous BCd dispersion (0.48 mg/mL) when adding a solution of chitosan (CS) in 2% acetic acid (0.48 mg/mL).

The presence of carboxylate groups on the BCd surface is confirmed by the negative ζ-potential of the BCd dispersion (−15 mV). To study the interaction of BCd with CS, we titrated the aqueous dispersion of BCd with a solution of CS in 2% acetic acid and measured the ζ-potential of the resulting particles (Figure 2). The change in the sign of the ζ-potential indicates the interaction between the negatively charged BCd nanofibers and the positively charged CS molecules. 

The presence of these charges promotes the mutual repulsion of BCd nanofibers and prevents their aggregation during dispersion in water, which further ensures uniform distribution of BCd in the CS matrix.

### 2.2. Wide-Angle X-ray Scattering

Cellulose is semicrystalline polymer with two different crystal structures in the native state, namely Iα and Iβ. Cellulose Iα crystallizes in a triclinic unit cell, space group P1 with a = 6.717 Å, b = 5.962 Å, fiber repeat c = 10.400 Å, α = 118.08°, β = 114.80°, γ = 80.37°, and Iβ in a monoclinic unit cell, space group P21 with a = 7.784 Å, b = 8.201 Å, fiber repeat c = 10.38 Å, γ = 96.5° [33].

**Figure 3:**
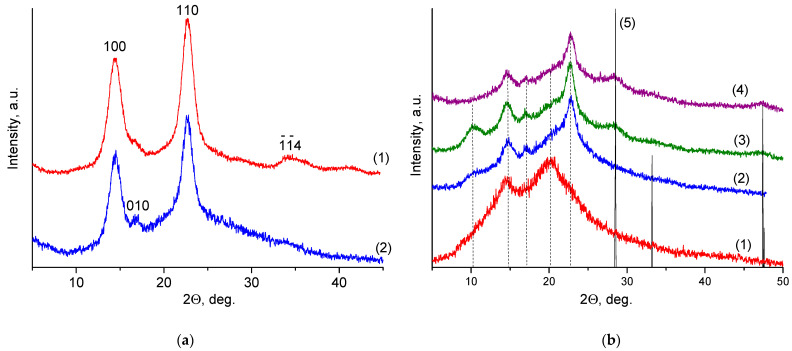
Wide-angle X-ray scattering patterns of: (**a**) BC (1), BCd (2); (**b**) CS (1), CS(80)-BCd(20) (2), CS(80)-BCd(20)-CeONP(4) (3), Cs(80)-BCd(20)-CeONP(8) (4), CeO_2_ ICDD PDF card #34-394 (5).

Figure 3a shows the wide-angle X-ray scattering (WAXS) pattern obtained for the BC film, which is typical of cellulose I. Three diffraction peaks at 2θ = 14.5°, 16.6°, and 22.7° are attributed to the (100), (010), and (110) planes of cellulose Iα (triclinic), or the $\left(1\overset{-}{1}0\right)$, (110), and (200) planes of cellulose Iβ (monoclinic) [34]. Because the positions of these peaks corresponding to two allomorphic phases are too close together, it is difficult to distinguish between them. Usually BC consists mainly of the Iα modification.

It should be noted that the two WAXS patterns are similar to each other, but the X-ray diffraction peaks revealed for the BCd film have a lower intensity compared to those for BC. In other words, the intensity of the amorphous halo in the BCd pattern is much higher than that in the BC pattern. The reason is believed to be a partial amorphization (i.e. a decrease in a degree of crystallinity) of BCd. 

It should also be noted that in the BCd film, an increase in the intensity of the (010) peak and a simultaneous disappearance of the $\left(\overset{-}{1}\overset{-}{1}4\right)$ peak can be observed [35]. This means that some changes in the texture (i.e., preferential orientation of the crystallites) take place in the BCd film compared to the BC one. It is also clearly seen that the X-ray diffraction peaks detected in the BCd sample are narrower than those in the BC film. This could be explained by the fact that the crystallites of the BCd film are less defective.

The diffractogram of CS (Figure 3b(1)) shows reflexes at around 15° and 22°, corresponding to the anhydrous polymorphic modification of CS [36]. The CS(80)-BCd(20) film (Figure 3b(2)) shows a weak reflex in the region of 2θ = 10.2° and reflexes at 14.8° and 17.0° as well as a blurred reflex in the angle range of 20–23°. Thus, the diffractogram of the control CS(80)-BCd(20) sample contains both the reflexes typical for the hydrated polymorphic modification of CS (10°, 15°, and 20°) [36] and the reflexes typical for BCd.

The diffractogram of CS(80)-BCd(20)-CeONP(4) (Figure 3b(3)) shows a more intense reflex in the 10° region compared to the control, corresponding to the hydrated polymorphic modification of CS. Increasing the CeONP content in CS(80)-BCd(20)-CeONP(8) (Figure 3b(4)) leads to the disappearance of this reflex (anhydrous CS polymorphic modification).

For CS(80)-BCd(20)-CeONP(4) and Cs(80)-BCd(20)-CeONP(8) (Figure 3b(3,4)) both reflexes are observed typical of the control film and reflexes in the regions 2θ = 28.7°, 33°, and 47.5° corresponding to reflexes of the CeO_2_ crystal lattice planes (111), (200), (220), and (311), respectively (Cubic crystal structure of fluorite: ICDD PDF card #34-394, data from National Institute of Standards and Technology, Gaithersburg, MD, USA) [37].

### 2.3. Swelling Properties

A comparative study of swelling properties of the composite films (Table 1) indicates that the introduction of BCd into the CS film promotes an increase in the swelling degree in water, with the swelling degree increasing with the increasing BCd content (Table 1, the CS(95)-BCd(5) and CS(80)-BCd(20) samples). The incorporation of 4% CeONPs into the composites also leads to an increase in the swelling degree in water compared to the control films. However, increasing the CeONP content up to 8% leads to a decrease in the swelling degree of the nanocomposite film both in water and in saline solution. The changes in the hydrophilicity of the nanocomposite films are due to the modification of the nanocomposite structure and the change in the nature of the intermolecular interactions.

### 2.4. Mechanical Properties

To compare the contribution of both polysaccharides to the nanocomposite film properties, the mechanical properties of individual CS and BCd films were investigated (Table 2). The BC-based film is 2.5 times superior to the CS film in Young’s modulus value, but it is almost 10 times inferior in deformation value. Therefore, the introduction of BCd into the CS films and the increasing concentration of BCd in the CS-BCd composition leads to a sequential increase in the stiffness of the material and a decrease in the elongation at break (Table 2). At the same time, due to the increase in Young’s modulus, the decrease in ε_b_ does not lead to a decrease in the strength of the nanocomposite film with increasing BCd content.

The incorporation of CeONPs into the polysaccharide matrices does not significantly change the Young’s modulus of the films formed (Table 2). This fact shows that the formation of such nanocomposites does not lead to a significant strengthening of the intermolecular bonds in the material. At the same time, the X-ray diffraction data show certain interactions of CeONPs with the polysaccharide composite film. These apparently contradictory results indicate that the introduction of nanoparticles into the polymer composition leads to a rearrangement of the intermolecular binding, but that this rearrangement has a complex nature. On the one hand, some interactions of CS macrochains with the surface of nanoparticles are formed, which may have the function of interchain cross-links that increase the stiffness of the material. However, the introduction of nanoparticles ~3 nm in size [38] into the polymer matrix leads to a local increase in interchain distances in the polymer matrix and, consequently, to a certain decrease in the density of intrinsic intermolecular interactions in the polymer base of the composite. Apparently, these two opposite processes balance each other in the formation of the nanocomposite material.

We can also note that the incorporation of nanoparticles into the polysaccharide matrix, in both concentrations used in this work (4% and 8%), leads to a decrease in the ultimate deformation typical of polymer-inorganic nanocomposites.

**Figure 4:**
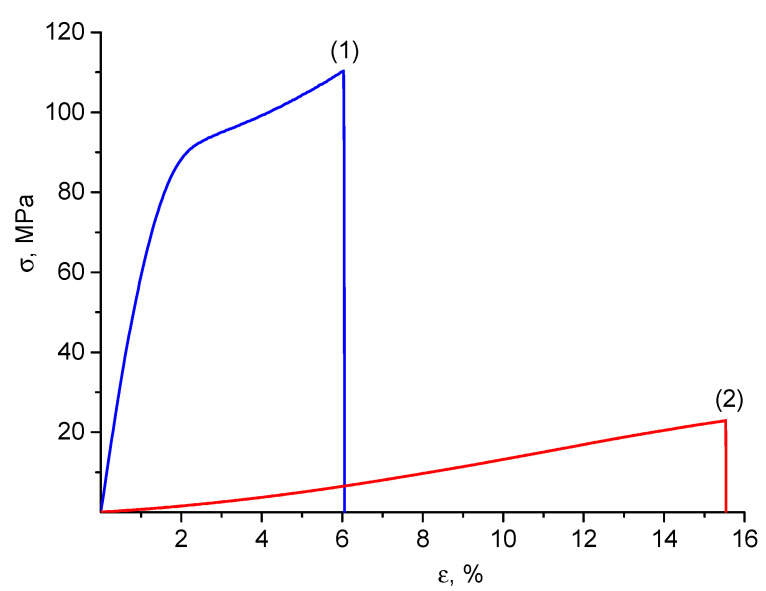
Stress–strain curves of the CS(80)-BC(20)-CeONP(8) film in dry (1) and swollen (2) states.

It is of special interest to characterize the mechanical properties of the developed nanocomposite material in the swollen equilibrium state. Indeed, for biomedical applications, film materials are used not in the dry but in the wet form. The mechanical tests of the swollen film CS(80)-BCd(20)-CeONP(8) with ~470% water relative to the weight of the dry material (Table 2 and Figure 4) showed that even in this state the nanocomposite material is superior in strength to, for example, polyethylene films and is suitable for practical use.

### 2.5. Thermal Analysis

**Figure 5:**
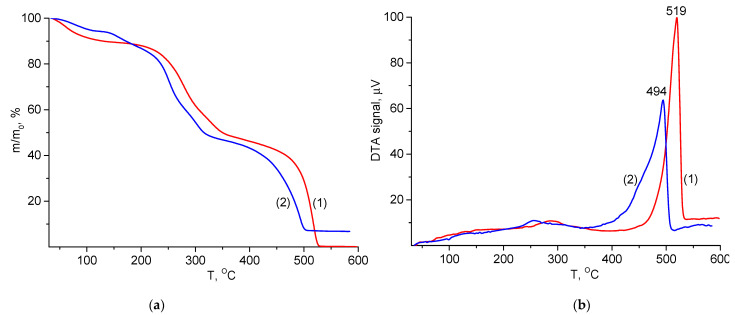
TGA (**a**) and and differential thermal analysis (DTA) (**b**) curves of (1), CS(80)-BCd(20), (2), CS(80)-BCd(20)-CeONP(8) films in air atmosphere.

The thermal degradation of CS(80)-BCd(20) and CS(80)-BCd(20)-CeONP(8) in air proceeds in two stages (Figure 5a), which is typical of many polysaccharides [39,40]. In the first stage (200–350 °C), both the control sample and the CS(80)-BCd(20)-CeONP(8) composite lose ~40% of their weight. At the second high temperature stage (400–550 °C), the complete decomposition of the CS-BCd film occurs with the transition of thermal-oxidative degradation products into the gas phase, and the weight of the nanocomposite decreases to a residual value of 7.3%, after which it remains constant during further heating. This residual mass corresponds to the concentration of CeO_2_ in the film. Taking into account the concentration of water in the material (weight loss at the initial stage of heating to 100–150 °C), the content of oxide nanoparticles is 8.1%.

The thermo-oxidative degradation processes in the first, low-temperature step correspond to the initial stage of pyrolysis when dehydration, depolymerization, and decomposition of acetylated and deacetylated units occur simultaneously. At this stage, pyrolysis causes the random cleavage of glycosidic bonds, followed by further decomposition, resulting in the formation of C2, C3, and C6 fatty acids, including acetic acid and butyric acid. The further process of deep destruction of materials takes place at the high temperature stage, up to 500–550 °C. At this stage, the oligomeric and monomeric products formed at the first stage decompose in an oxygen-containing atmosphere resulting in the formation of gaseous substances [41]. A similar pattern of thermo- oxidation of BCd was reported in our previous studies [37].

The thermogravimetric analysis (TGA) results show that the incorporation of CeONPs into the polysaccharide matrix did not lead to a significant change in the temperature characteristics of the thermo-oxidative degradation of the material over a wide temperature range (up to 450–550 °C). In particular, the temperature resistance indices of CS-BCd and CS-BCd-CeONP films (Table 3) were almost identical. It was only in the last stage of degradation (in the region above 450 °C) that an increase in degradation of the CeONPs containing nanocomposite was observed compared to the original CS-BCd film (Figure 5a).

This trend is well illustrated by the differential thermal analysis (DTA) curves of the degradation processes of CS-BCd and CS-BCd-CeONP films (Figure 5b). The heat release peak for the CS-BCd-CeONP film (maximum at 521 °C) is shifted by 10 °C to lower temperatures with respect to the heat release maximum for the CS-BCd film. It should also be noted that the DTA curves show a significant difference in the energy characteristics of the material degradation occurring in two stages of thermal-oxidative degradation (low and high temperature). The first stage proceeds without significant heat release: the DTA curves show a low exothermic effect with the maximum intensity of heat release at 282 °C, and in the second stage, an intense exothermic peak is registered (Figure 5b).

### 2.6. Scanning Electron Microscopy

**Figure 6:**
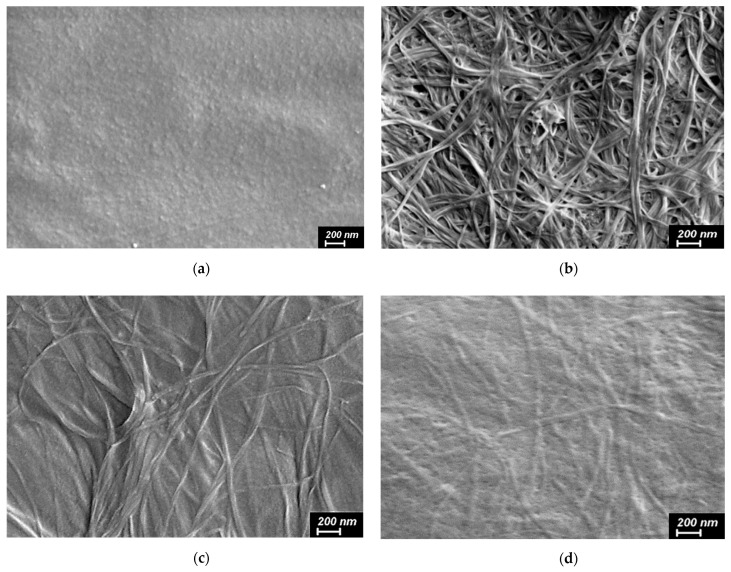
Scanning electron microscopy (SEM) images of the surfaces of the CS film (**a**), dried dispersion of BCd (**b**), the CS(80)-BC(20) (**c**) and BCd-CS-CeONP(8) (**d**) films.

The CS film shows a uniform surface, while BCd shows a random network of dried nanofibers with a thickness of 20–30 nm (Figure 6). The surface morphology of the control CS(80)-BCd(20) film is characterized by a random distribution of BCd nanofibers in the CS matrix. In the CS(80)-BCd(20)-CeONP(8) film, the BCd nanofibers are less visualized than in the CS(80)-BCd(20) film, which may indicate a stronger interaction between the BCd nanofibers and the CS-CeONP.

**Figure 7:**
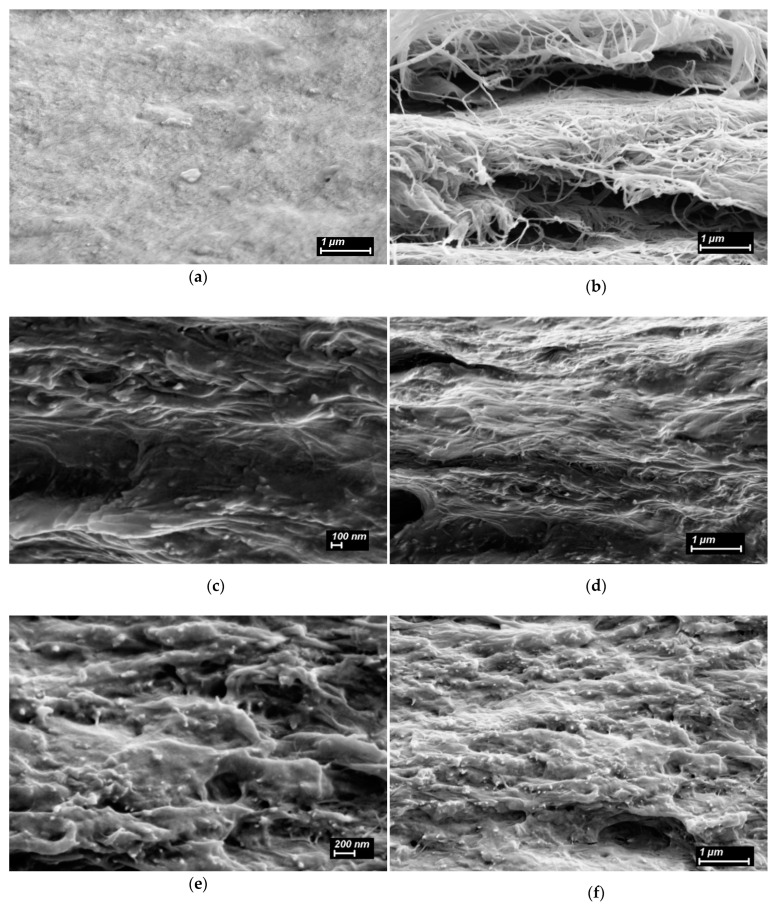
SEM images of cryocleaved surfaces of the CS film (**a**), dried dispersion of BCd (**b**), the CS(80)-BC(20) (**c**,**d**) and CS(80)-BCd(20)-CeONP(8) (**e**,**f**) films.

The scanning electron microscopy (SEM) image of the CS film cryocleavage (Figure 7) shows no ordered fragments. At the same time, the BCd film contains layers of ordered nanofibers. The ordered BCd nanofibers were observed in the cleavage of CS(80)-BCd(20) films, and they were covered with CS solution and formed a bonded structure, which may further indicate the interaction between CS and BCd nanofibers. The incorporation of CeONPs (the CS(80)-BCd(20)-CeONP(8) sample, Figure 7e,f) changes this pattern: the BCd nanofibers are not visible and the SEM shows a denser packing. The white dots in the SEM images of the cryocleavages of the CS-BCd-CeONP composites (Figure 7c–f) are the broken ends of the BCd nanofibers and the fracture surfaces of these nanofibers formed during the cryocleavage of the materials.

**Figure 8:**
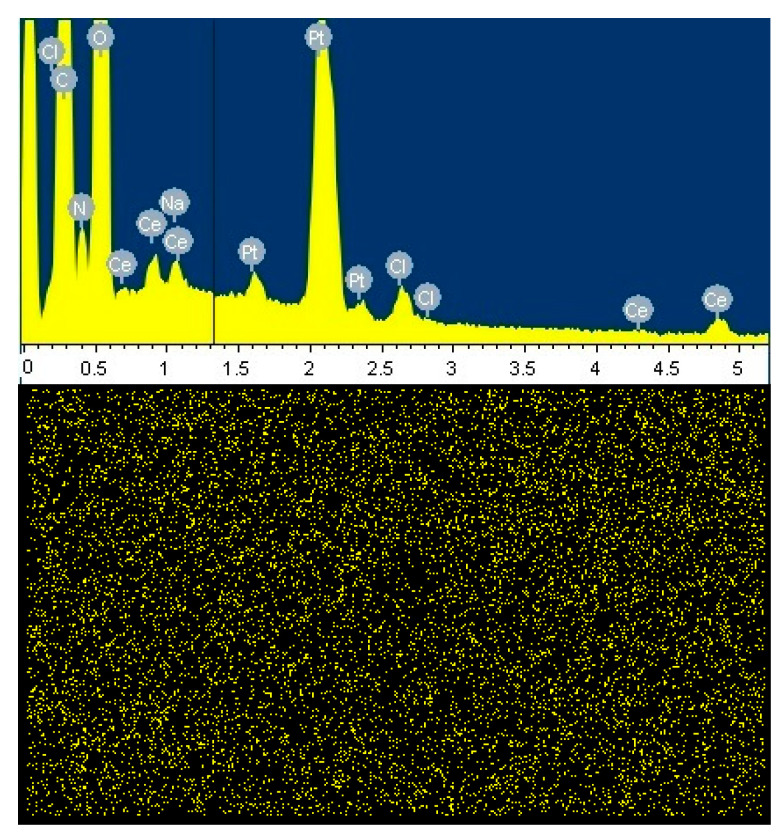
Energy dispersive X-ray spectrum and map of Ce distribution on the surface of a CS(80)-BCd(20)-CeONP(8) film.

In addition, the surface of the samples was examined using energy-dispersive X-ray spectroscopy (EDX), which allows the presence and distribution of chemical elements in a sample to be determined (in this case, the distribution of cerium). EDX confirmed the incorporation of cerium-containing species into CS(80)-BCd(20)-CeONP(8). Cerium distribution maps showed the uniform distribution of this element in the samples (Figure 8).

### 2.7. Culture of Multipotent Mesenchymal Stem Cells

#### 2.7.1. Quantitative Analysis

The number of isolated MSCs and cells in the flat colonies was significantly higher in CS(80)-BCd(20)-CeONP(8) compared to the control (*p* < 0.05). In the other experimental groups, no significant differences in the number of nuclei were found compared to the control (*p* > 0.05). The number of nuclei on the surface of CS(80)-BCd(20)-CeONP(8) was significantly higher than in CS(95)-BC(5)-CeONP(4), while there were no significant differences with CS(80)-BCd(20)-CeONP(4) (*p*>0.05) (Table 4). 

There were no significant differences in the number of spheroid colonies in the experimental groups compared to the control and each other (*p* > 0.05). The spheroids in CS(95)-BCd(5)-CeONP(4) and CS(80)-BCd(20)-CeONP(8) were larger in size compared to the control and the other experimental groups (*p* < 0.05). In addition, there were no significant differences in the size of spheroidal colonies in control and CS(80)-BCd(20)-CeONP(4) (*p* > 0.05) (Table 4).

#### 2.7.2. Qualitative Analysis

On coverslips, the cells were evenly arranged and spread on the glass surface, forming a confluent/sub-confluent monolayer similar to syncytium, and had a typical elongated shape with multiple processes; in addition, some cells were in the process of division. Longitudinal linear structures stained red, actin microfilaments, were clearly seen in the cells.

**Figure 9:**
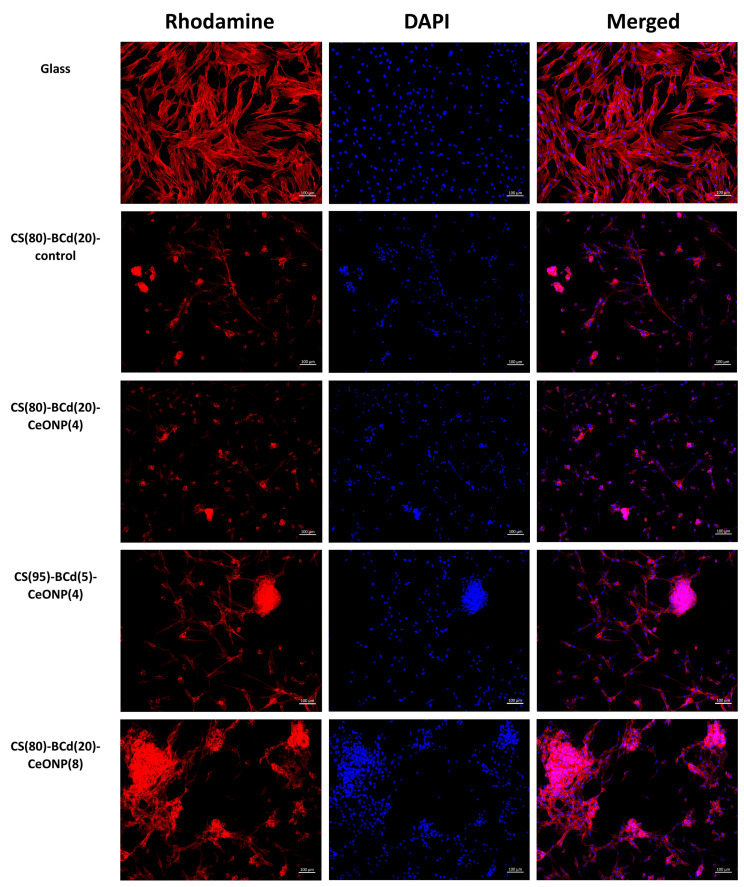
MSCs adhered to the surface of glasses and CS-BCd composite films. Staining of cell cytoskeleton fibrillar actin with Rhodamine-channel fluorochrome visualization, staining of cell nuclei with DAPI (4,6-diamidino-2-phenylindole), combined two-channel image. Magnification ×100.

**Figure 10:**
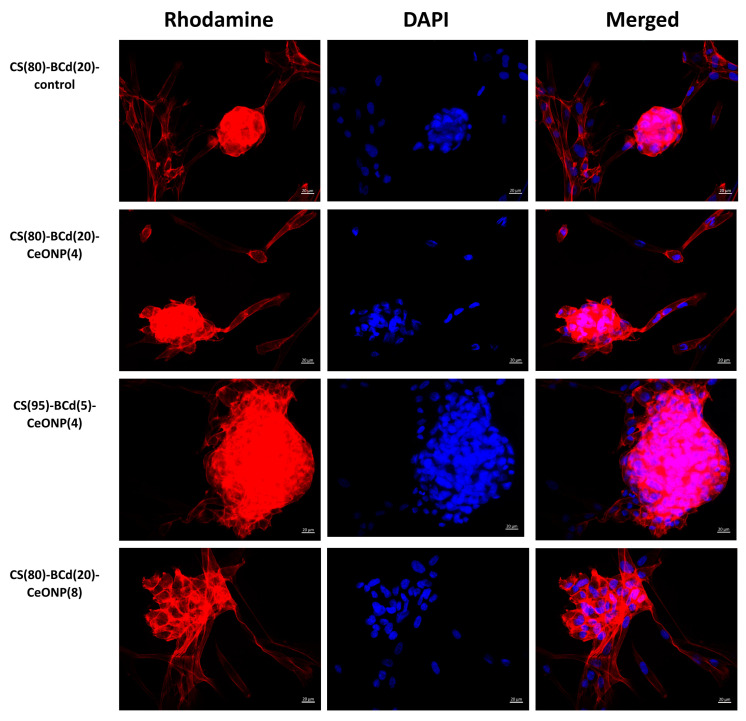
Spheroid colonies on the surface of the CS-BCd composite films. Staining of cell cytoskeleton fibrillar actin with Rhodamine-channel fluorochrome visualization, staining of cell nuclei with DAPI, combined two-channel image. Magnification ×400.

In all experimental groups, the cells were irregularly arranged on the surface of the samples in the form of single cells, flat, and spherical colonies (Figure 9). Some of the solitary cells had a typical elongated shape with processes; another part had a round/near round shape with or without cytoplasmic protrusions at the periphery (probably in the state of blebbing). Flat colonies were visualized as large or smaller groups of cells connected by syncytial-like processes or as star-shaped colonies. Spheroid colonies were more often rounded/oval in shape, less often oblong/non-rectangular in shape. Some spheroids showed evidence of cell migration along the periphery of the colonies in the form of individual cells protruding along the periphery without connection to surrounding cells/colonies (+), cell bridges with other flat/spheroidal colonies (++), or broad fusion with flat colonies (spheroid-to-monolayer transition). No evidence of cell migration was observed in some spheroid colonies (Figure 10, Table 5).

In CS(80)-BCd(20)-CeONP(4) and CS(80)-BCd(20) (as a control), multiple separately arranged cells of both typical elongated shape and rounded/nearly rounded shape were detected (Figure 9). Flat stellate and syncytial colonies consisted of a small number of cells. Single dividing MSCs were detected among the separated cells as well as within the flat colonies. Most of the spheroid colonies showed signs of cell migration: in CS(80)-BCd(20) and CS(80)-BCd(20)-CeONP(4), migration was observed as single cells at the periphery of the spheroids and single bridges with the surrounding cells/colonies. Single spheroidal colonies without cell migration were visualized on the surface of the above sample groups (Figure 10, Table 5).

In CS(95)-BCd(5)-CeONP(4), individually arranged cells of typical elongated shape and rounded/nearly rounded cells were observed in small numbers (Figure 9). Most of the adherent cells formed flat syncytium-like colonies, which occupied a larger area compared to the colonies of CS(80)-BCd(20). Single, dividing MSCs were detected among the cells in the flat colonies. Most of the spheroidal colonies showed signs of cell migration in the form of cell bridging and fusion with the flat colonies. Single spheroidal colonies without signs of cell migration were visualized on the surface of the above samples (Figure 10, Table 5).

In CS(80)-BCd(20)-CeONP(8), the separately arranged cells of typical elongated shape and rounded/nearly rounded cells were detected in small numbers (Figure 9). Most of the adherent cells formed flat syncytium-type colonies, which occupied a larger area compared to the colonies in the other samples. Among the cells in the flat colonies, we detected individual MSCs in the process of division. Most of the spheroid colonies showed signs of cell migration in the form of multiple cell bridges and fusion with the flat colonies. Single spheroidal colonies without signs of cell migration were visualized on the surface of the above samples (Figure 10, Table 5).

Therefore, the greatest number of adherent cells located separately and as part of planar colonies (outside of spheroids) was detected on the surface of CS(80)-BCd(20)-CeONP(8). In addition, the number of isolated cells (outside the planar and spheroidal colonies), including those in a state of apoptosis, was greater on the surface of both the control CS(80)-BCd(20) and CS(80)-BCd(20)-CeONP(4) samples. 

On the surface of CS(95)-BCd(5)-CeONP(4) and CS(80)-BCd(20)-CeONP(8), most adherent cells were found in flat and spheroidal colonies. Spheroidal colonies were observed in all samples. No significant differences were found in the number of spheroidal colonies on the surface of the samples, with the colonies in CS(95)-BCd(5)-CeONP(4) and CS(80)-BCd(20)-CeONP(8) being larger. Maximum cell migration was observed in CS(95)-BCd(5)-CeONP(4) and CS(80)-BCd(20)-CeONP(8). Finally, the proliferation of adherent cells with primary formation of squamous colonies was more pronounced in CS(80)-BCd(20)-CeONP(4). CS(80)-BCd(20)-CeONP(8) showed the best adhesion properties and biocompatibility.

In summary, cell adhesion, spreading, proliferation, differentiation, and function are highly dependent on scaffold properties such as mechanical properties, chemical structure, nano- and microtopography, surface charge, porosity, wettability, etc. [42,43]. CS-based scaffolds have many advantages, including biocompatibility, biodegradability, and non-toxicity. However, CS films have high deformability and insufficient strength in the swollen state. The incorporation of BCd into the CS film not only improves the mechanical properties, but also may contribute to the regulation of the surface charge due to the ionic bonding of negatively charged BCd nanofibers with positively charged protonated amino groups of CS. In addition, CeONPs are known to influence intracellular signaling pathways and overall cellular metabolism [44,45].

The developed composite containing CS, BCd, and CeONPs showed an altered topography with a stronger interaction between CS, BCd nanofibers, and CeONPs. Apparently, the altered topography improved the adhesive properties of the composite scaffolds. The combination of complex physicochemical interactions, including hydrophobic, coulombic, and van der Waals forces between the cell membrane and the material surface, occurs at the very beginning of the cell adhesion process [43,46]. Thus, the presence of negatively charged BCd nanofibers and positively charged CS molecules not only promotes the mutual repulsion of BCd nanofibers and prevents their aggregation during dispersion in water, but also influences the charge of the material and the initiation of the cell adhesion process.

## 3. Methods and Materials

### 3.1. Polysaccharides

The CS sample (Ennagram, Pantin, France) from crab shells with a molecular weight of 160,000 (capillary viscometry) and a degree of deacetylation of 0.80 (conductometric back titration) was used [47]. 

BC was produced by *Kommagateibacter xylinus* (acetic acid bacteria, a VKM-880 strain) in aqueous solutions containing 2 wt% of glucose, 0.3 wt% of yeast extract, and 2 wt% of ethanol at 30 °C for 14–21 days [48,49]. BCs were isolated by boiling in 6% NaOH followed by multiple rinses with water to neutral pH. The as-prepared BC samples were gel-like pellicles up to 25 mm thick. The pellicles were partially dehydrated using a hand press. The pressed BC was disintegrated in a high-speed blender (15,000 rpm, 15 min) in an aqueous medium (300 mL of water per 1 g of dry BC); the resulting sample was a dispersion of disintegrated BC (BCd).

### 3.2. Preparation of Composite Films

The CS film (control) was prepared by extruding a 3% solution of CS in 2% acetic acid through a spinneret onto a glass substrate, followed by drying at room temperature and further heating at 80 °C for 4 h.

The detailed protocol of BCd film formation has been described elsewhere [50].

CeONPs used in the experiments were synthesized according to the protocol described elsewhere [38]. CeONPs had a ζ-potential of –25.0 mV and a hydrodynamic diameter (D_h_) of 9 nm.

CS-stabilized CeONPs were prepared according to the procedure described elsewhere [32]. CS-coated CeONPs had a ζ-potential of +20.3 mV and a D_h_ of 244 nm.

The film-casting mixtures were prepared by mixing a 2% solution of CS in 2% acetic acid, a 0.3% dispersion of BCd, and a dispersion of CeONPs. The resulting mixtures were homogenized by mechanical stirring for 1 h. Films were prepared from the composite mixtures by dry casting; the mixtures were extruded through a spinneret onto a glass substrate and dried at room temperature (film thickness was 30–40 μm). The composite films were then heated at 80 °C for 2 h, which caused them to lose their solubility in water [51]. The composite films of different compositions were prepared (the CS/BCd ratio was 80%/20% or 95%/5%); the amount of CeONPs (4% or 8%) was calculated from the total polymer mass. The resulting samples were as follows: CS(80)-BCd(20)-CeONP(4); CS(80)-BCd(20)-CeONP(8); CS(95)-BCd(5)-CeONP(4); and control films: CS(80)-BCd(20); CS(95)-BCd(5). 

### 3.3. Characterization of Composites

FTIR spectra of the films were recorded on a Vertex 70 IR Fourier spectrometer (Bruker Optik, Ettingen, Germany). A Pike MIRacle attenuated total reflection sampling accessory (Pike Technologies, Madison, WI, USA) with a ZnSe working element was used to preserve the structure of the films. In the registration of the spectra, a correction was introduced that takes into account the penetration depth depending on the wavelength.

The ζ-potential of BCd nanofibers was determined using a Photocor Compact-Z instrument (Photocor Ltd., Moscow, Russia) at a laser wavelength of 659 nm and a detection angle of 90°.

To study the interaction between BCd and CS, a solution of CS in 2% acetic acid (C = 0.48 mg/mL) was added dropwise to a dispersion of BCd (2.5 mL, C = 0.48 mg/mL) and the ζ-potential of the resulting systems was measured.

The equilibrium swelling degree of the films (the swelling degree after 24 h exposure to water) was determined by the gravimetric method.

The resulting films were characterized by SEM using a SUPRA-55VP scanning electron microscope (Zeiss, Oberkochen, Germany) and by WAXS using a D8 DISCOVER X-ray diffractometer (Bruker, Karlsruhe, Germany) with CuKα radiation.

SEM images were obtained using both a secondary electron detector and a backscattered electron detector. To visualize the distribution of CeONPs in the samples, the films were frozen and split in liquid nitrogen, then glued to a conductive tape, sputtered through a thin layer of platinum, and analyzed EDX to obtain the maps of Ce distribution. The elemental maps were obtained using an EDX-Max 80 mm^2^ detector (Oxford Instruments, Oxford, UK). The analysis was performed over the whole visible range of the samples.

An AG-100kNX Plus setup (Shimadzu, Kyoto, Japan) operating in uniaxial extension mode was used to investigate the mechanical properties of the films. Strip-like samples (2 × 20 mm) were stretched at room temperature at a rate of 2 mm/min according to the requirements of ASTM D638. The stress–strain curves of the samples were recorded during the tests. Young’s modulus (E), yield stress (σ_b_), and ultimate strength (ε_b_) were determined.

TGA and DTA were carried out to determine the residual water concentration in the films, the content of cerium oxide in the nanocomposite materials, and to characterize the effect of the nanofiller on the thermal properties of the composite films. TGA curves were used to determine the thermal stability indices of the samples, τ5 and τ10 (the temperatures at which a polymer or composite loses 5% and 10% of its initial weight, respectively, as a result of thermal destruction processes). A DTG-60 thermal analyzer (Shimadzu, Kyoto, Japan) was used and samples (~5 mg) were heated in air to 600 °C at a rate of 5 °C/min.

### 3.4. Biocompatibility Testing

Adhesive properties of the materials were investigated using MSCs derived from the adipose tissue of healthy donors. The study was conducted in accordance with the Declaration of Helsinki, and approval was obtained from the local Ethical Committee of the Almazov National Medical Research Centre (№12.26/2014; 1 December 2014). Written informed consent was obtained from all subjects prior to adipose tissue biopsy. The study was conducted as previously described [10,42,52].

The biocompatibility of cell culture with nanocomposite samples was investigated using CS(80)-BC(20) and 12 mm diameter coverslips as controls. Cell culturing was performed in α-MEM medium supplemented with 10% fetal bovine serum, 1% L-glutamine, and 1% penicillin/streptomycin solution in a CO_2_ incubator at 37 °C and 5% CO_2_.

Samples of 12 × 8 mm materials (depending on the size of the well) were kept in phosphate-buffered saline (PBS) with the addition of 2% penicillin/streptomycin solution for 30 min, followed by three washes with PBS. The coverslips were sterilized in 70% ethanol for 10 min, followed by three washes with PBS. The samples and coverslips were then placed in the wells of a 24-well plate. Then, 1 mL of medium was added to the wells and incubated for 24 h in a CO_2_ incubator (to ensure uniform distribution of the components of the medium in the structure of the samples). After 24 h, the medium was removed and a suspension of MSCs at a concentration of 50,000 cells/mL was added to the wells and co-cultured for 72 h in a CO_2_ incubator. The experiment was performed in triplicate.

After 3 days, the samples and coverslips were transferred to the wells of a new plate, washed with PBS, and fixed with 4% paraformaldehyde solution for 10 min. 

After fixation, samples and coverslips were washed from paraformaldehyde with PBS and stained with rhodamine-labeled phalloidin according to a previously developed protocol. According to the protocol, samples and coverslips containing cells were first treated with 0.05% Triton X-100 solution for 3 min and then washed three times with PBS. Next, a solution of rhodamine-conjugated phalloidin at a dilution of 1:500 in 1% fetal bovine serum solution in PBS was added to the wells, incubated for 20 min at room temperature, and then washed five times with PBS. Finally, cell nuclei were stained with DAPI (4,6-diamidino-2-phenylindole) at a dilution of 1:40,000, incubated for 40 s, and then the samples were thoroughly washed from DAPI with PBS.

After staining, the samples were stored in PBS in the dark at +4 °C. The coverslips containing cells from the control wells were mounted on glass slides with mounting medium and stored in the dark at room temperature.

Fluorescence microscopy with qualitative and quantitative analysis of adherent cells was performed on the stained MSCs on film samples and glasses. An Axiovert inverted fluorescence microscope (Zeiss, Oberkochen, Germany) and a compatible Canon camera were used to visualize the cells. Pieces of material containing cells were placed between two coverslips. DAPI fluorescence was recorded using the DAPI channel, and rhodamine-phalloidin fluorescence was recorded using the rhodamine channel. Ten different fields of view were photographed at ×100 and ×400 magnifications for each technical replicate. Quantitative analysis consisted of counting the number of nuclei on the surface of the film samples (separately located cells and cells in flat colonies in fields of view without spheroids), counting the number of spheroid colonies per unit area (1 mm^2^), and estimating spheroid size (maximum longitudinal size). The number of nuclei in spheroids was not assessed because it is technically impossible to accurately count DAPI-positive signals in colonies with a 3D structure. The morphology of cells and their colonies was assessed qualitatively by staining the cytoskeleton, the type of colonies (flat colonies, spheroids), the presence and extent of cell migration along the periphery of spheroid colonies (+—single protruding cells along the periphery of spheroid not in contact with surrounding cells, ++—single cell bridges with surrounding cells, +++—fusion with surrounding flat colonies and transition of spheroid into monolayer). Statistical processing of the obtained data was performed with the GraphPad Prism 6.01 software (GraphPad Software, San Diego, CA, USA) using the non-parametric Mann–Whitney U criterion. The results were presented as mean ± standard error.

## 4. Conclusions

In this work, biocompatible CS-based composites containing a combination of two types of nanofillers, namely BCd nanofibers and CeONPs, were developed. The mechanical disintegration of the pressed gel film of BC leads to a change in the structural organization of the polymer and is accompanied by the formation of –COO^−^ groups on the surface of the resulting BCd nanofibers. The results of titration of the aqueous dispersion of BCd with a solution of CS in acetic acid suggest the possibility of interaction of CS with BCd nanofibers. CeONPs precoated with CS to impart a positive charge to the particles showed high compatibility with the polymer matrix and uniform distribution throughout the volume of the material.

The influence of the amount of introduced nanofillers with different architectures (BCd nanofibers and CeONPs) on the structure and properties of the composites was investigated. An increase in film stiffness was observed as a result of reinforcing the CS matrix with BCd nanofibers: the Young’s modulus increased from 4.55 to 6.3 GPa with the introduction of 5% BCd. A further increase in Young’s modulus of 6.7 GPa and a significant increase in film strength (22% increase in yield stress compared to the CS film) were observed when the BCd concentration was increased to 20%. The incorporation of CeONPs into the polysaccharide matrices has no significant effect on the mechanical properties of the films, while the X-ray diffraction data indicate the interaction of CeONPs with the polymeric components of the composite and the nature of this interaction depends on the amount of nanoparticles introduced. The introduction of 4% CeONPs is accompanied by an increase in the hydrophilicity of the nanocomposite, an increase in the CeONP concentration up to 8% contributes to a change in the structural organization of the nanocomposite, accompanied by a decrease in the swelling degree and the formation of a denser structure. The CS(80)-BCd(20)-CeONP(8) composite exhibits good strength in both dry and swollen states.

All nanocomposite matrices were found to be biocompatible. The CS(80)-BCd(20)-CeONP(8) composite showed the best biocompatibility and improved cell adhesion to the scaffold surface. Thus, the positive effect of the introduction of BCd and CeONPs on the properties of the CS-based nanocomposite film was demonstrated, and the resulting films were found to be promising for use as a matrix material for MSC culture. In addition, the results obtained suggest the possibility of successfully using the developed materials for the preparation of wound dressings.

## Figures and Tables

**Table 1:** The swelling degree of the CS and composite films.

Sample	Swelling Degree in Water (g/g)	Swelling Degree in 0.9% NaCl Solution, (g/g)
CS	2.6	-
CS(80)-BCd(20)	10.8	6.2
CS(80)-BCd(20)-CeONP(4)	13.0	6.8
CS(80)-BCd(20)-CeONP(8)	4.7	2.9
CS(95)-BCd(5)	6.6	5.1
CS(95)-BCd(5)-CeONP(4)	10.2	8.9

**Table 2:** Mechanical properties of the polysaccharides and nanocomposite films.

Sample	E (GPa)	σ_b_ (MPa)	ε_b_ (%)
CS	4.55 ± 0.12	114 ± 5	30 ± 2
BCd	11.7 ± 0.3	460 ± 17	3.6 ± 0.3
CS(95)-BCd(5)	6.3 ± 0.4	110 ± 6	11 ± 2
CS(80)-BCd(20)	6.7 ± 0.3	140 ± 6	4.5 ± 0.6
CS(95)-BCd(5)-CeONP(4)	6.2 ± 0.5	118 ± 5	7 ± 1
CS(80)-BCd(20)-CeONP(4)	7.0 ± 0.4	111 ± 6	3.7 ± 0.4
CS(80)- BCd (20)-CeONP(8)	6.8 ± 0.2	110 ± 6	6 ± 1
CS(80)-BCd(20)-CeONP(8) in swollen state	(73 ± 3) × 10^−3^	22 ± 2	15 ± 2

**Table 3:** The thermogravimetric analysis (TGA) data of the polysaccharides and nanocomposite films.

Sample	τ5, °C	τ10, °C
CS(80)-BCd(20)	243	256
CS(80)-BCd(20)-CeONP(8)	239	255

**Table 4:** Adhesion of the human mesenchymal stem cells (MSCs) on the samples surface.

Sample	Adhered Cells, cells/mm^2^	Number of Spheroids	Spheroids’ Size, µm
CS(80)-BCd(20)—control	216 ± 16	6.6 ± 0.9	64 ± 7
CS(80)-BCd(20)-CeONP(4)	218 ± 22	8.2 ± 1.5	66 ± 6
CS(95)-BCd(5)-CeONP(4)	199 ± 10	6.7 ± 0.6	141 ± 17 *
CS(80)-BCd(20)-CeONP(8)	300 ± 33 *	6.8 ± 0.7	136 ± 12 *

Significance of differences compared to the control sample (Mann–Whitney), * *p* < 0.05

**Table 5:** Characterization of separately located cells and cell colonies on the surface of samples.

Sample	Separate cells		Type of Colonies	Cell Migration from Spheroids
	Elongated Cells	Rounded Cells		
Glass	multiple	-	flat colonies/monolayer	-
CS(80)-BCd(20)—control	multiple	multiple	flat colonies + spheroids	++
CS(80)-BCd(20)-CeONP(4)	multiple	multiple	flat colonies + spheroids	++
CS(95)-BCd(5)-CeONP(4)	single	single	flat colonies + spheroids	+++
CS(80)-BCd(20)-CeONP(8)	single	single	flat colonies + spheroids	+++

## Data Availability

Data available upon request.
